# Experimental Aristolochic Acid Nephropathy: A Relevant Model to Study AKI-to-CKD Transition

**DOI:** 10.3389/fmed.2022.822870

**Published:** 2022-05-04

**Authors:** Thomas Baudoux, Inès Jadot, Anne-Emilie Declèves, Marie-Hélène Antoine, Jean-Marie Colet, Olivia Botton, Eric De Prez, Agnieszka Pozdzik, Cécile Husson, Nathalie Caron, Joëlle L. Nortier

**Affiliations:** ^1^Laboratory of Experimental Nephrology, Faculty of Medicine, Université Libre de Bruxelles (ULB), Brussels, Belgium; ^2^Molecular Physiology Research Unit (URPhyM), Namur Research Institute for Life Sciences (NARILIS), University of Namur (UNamur), Namur, Belgium; ^3^Laboratory of Molecular Biology, Faculty of Medicine and Pharmacy, Research Institute for Health Sciences and Technology, University of Mons (UMONS), Mons, Belgium; ^4^Department of Human Biology & Toxicology, Faculty of Medicine and Pharmacy, Research Institute for Health Sciences and Technology, University of Mons (UMONS), Mons, Belgium

**Keywords:** aristolochic acid nephropathy, AKI-to-CKD transition, animal models, renal fibrosis, nephrotoxicants, aristolochic acids

## Abstract

Aristolochic acid nephropathy (AAN) is a progressive tubulointerstitial nephritis caused by the intake of aristolochic acids (AA) contained in Chinese herbal remedies or contaminated food. AAN is characterized by tubular atrophy and interstitial fibrosis, characterizing advanced kidney disease. It is established that sustained or recurrent acute kidney injury (AKI) episodes contribute to the progression of CKD. Therefore, the study of underlying mechanisms of AA-induced nephrotoxicity could be useful in understanding the complex AKI-to-CKD transition. We developed a translational approach of AKI-to-CKD transition by reproducing human AAN in rodent models. Indeed, in such models, an early phase of acute tubular necrosis was rapidly followed by a massive interstitial recruitment of activated monocytes/macrophages followed by cytotoxic T lymphocytes, resulting in a transient AKI episode. A later chronic phase was then observed with progressive tubular atrophy related to dedifferentiation and necrosis of tubular epithelial cells. The accumulation of vimentin and αSMA-positive cells expressing TGFβ in interstitial areas suggested an increase in resident fibroblasts and their activation into myofibroblasts resulting in collagen deposition and CKD. In addition, we identified 4 major actors in the AKI-to-CKD transition: (1) the tubular epithelial cells, (2) the endothelial cells of the interstitial capillary network, (3) the inflammatory infiltrate, and (4) the myofibroblasts. This review provides the most comprehensive and informative data we were able to collect and examines the pending questions.

## Introduction

For a long time, the common belief was that the long-term outcome of patients recovering from acute kidney injury (AKI) was benign. However, this previous paradigm has been progressively challenged as several epidemiological studies demonstrated that AKI significantly increases the risk of chronic kidney disease (CKD) onset (1–8). Indeed, severe or repeated AKI episodes initiate an exaggerated recovery process leading ultimately to rarefaction and sclerosis of glomeruli, atrophy and dilation of tubules, tubulointerstitial fibrosis and peritubular capillaries rarefaction, all being characteristic of CKD (9). All these epidemiological and clinical reports have raised the urgent need to determine whether the progression of this process could be stopped or at least delayed. In this regard, animal models represent a relevant tool to investigate the pathophysiological link between AKI and CKD and to assess therapeutic strategies (10). In addition to the classical models of ischemic reperfusion injury (IRI) and unilateral ureteral obstruction (UUO), experimental nephrotoxic approaches represent an attractive alternative.

Human aristolochic acid nephropathy (AAN) was formerly known as “Chinese herb nephropathy.” This tubulointerstitial nephritis was first reported in Belgian women after ingestion of herbal slimming remedies containing aristolochic acids (AA) (11). At this time, clinicians were amazed by the prominent interstitial fibrosis with a typical corticomedullary gradient and the tubular atrophy characterizing this nephrotoxicity (12). Later, AA metabolism was found to lead to DNA adducts formation (13). These specific AA-DNA adducts are known to induce carcinogenesis and mutagenesis by activation of *H-ras* proto-oncogene in rats and by *p53* gene mutation in humans finally leading to uretero-vesical cancers (14–18). Beside the Belgian outbreak, these nitrophenanthrene derivatives were also found responsible for thousands of cases of the so-called Balkan endemic nephropathy (19, 20). Finally, in China and Taiwan where traditional herbal medicines are still widely used, more than 300 cases have been described but this number is certainly underestimated as billions of Chinese and Taiwanese's patients could have been exposed to AA (21–24). Human AAN has been reproduced in several animal models including rabbits, mice and rats (25–29). Interestingly, a biphasic evolution of the tubulointerstitial lesions was demonstrated in our male Wistar rat model and in our mice models (26, 27, 30). The acute phase (several days following the beginning of AA exposure) is characterized by a necrosis of the proximal tubular epithelial cells (PTEC) concomitantly to a rise of plasma creatinine. This phase is rapidly followed by an inflammatory infiltrate along with a normalization of the biological parameters, reflecting an attempt of PTEC regeneration. Following the acute phase, interstitial fibrosis and tubular atrophy were the main hallmarks of the later chronic phase (already several days after starting AA exposure and persisting after cessation of AA intoxication). Regarding the marked interstitial fibrosis characterizing AAN, these models are of particular interest in order to study the AKI-to-CKD transition and the respective role of the different actors involved in this process.

## Major Cellular Mechanisms Underlying AKI-to-CKD Progression and Translation to AAN Models

**Figure 1:**
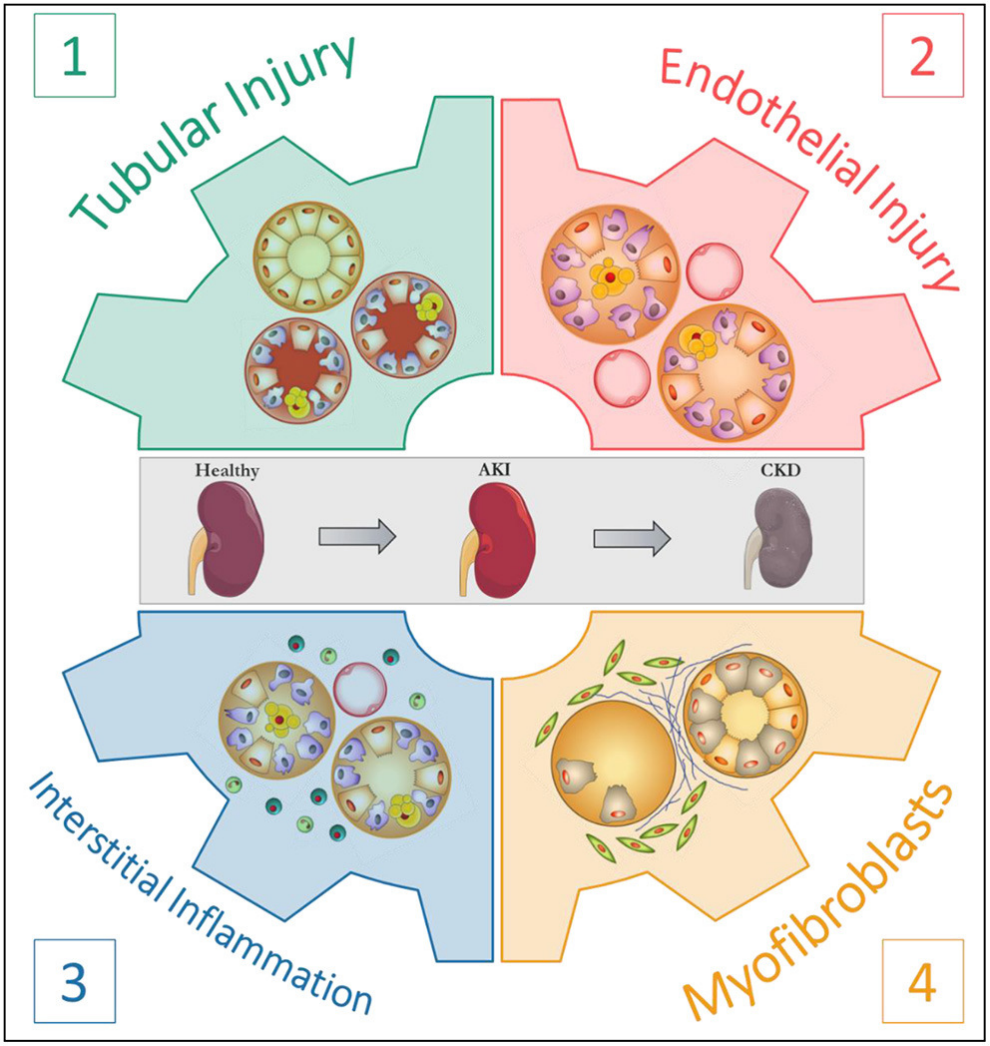
The four pivotal cellular responses in the progression of kidney disease. According to Eddy (31).

Clearly, mechanisms linking AKI to CKD are in part universal and independent of the initial aggression. Understanding the respective roles of the actors involved in the AKI-to-CKD transition is essential to develop prevention strategies. In this regard, Eddy proposed four pivotal cellular responses in the disease progression: (1) the response of tubular epithelial cells to the aggression; (2) the loss of endothelial integrity; (3) the interstitial inflammatory response and (4) the appearance of a new interstitial cell population, the myofibroblasts (Figure 1) (31). The following sections are dedicated to each of these major processes and their translation to rodent models of AAN.

### The Proximal Tubular Epithelial Cells

**Figure 2:**
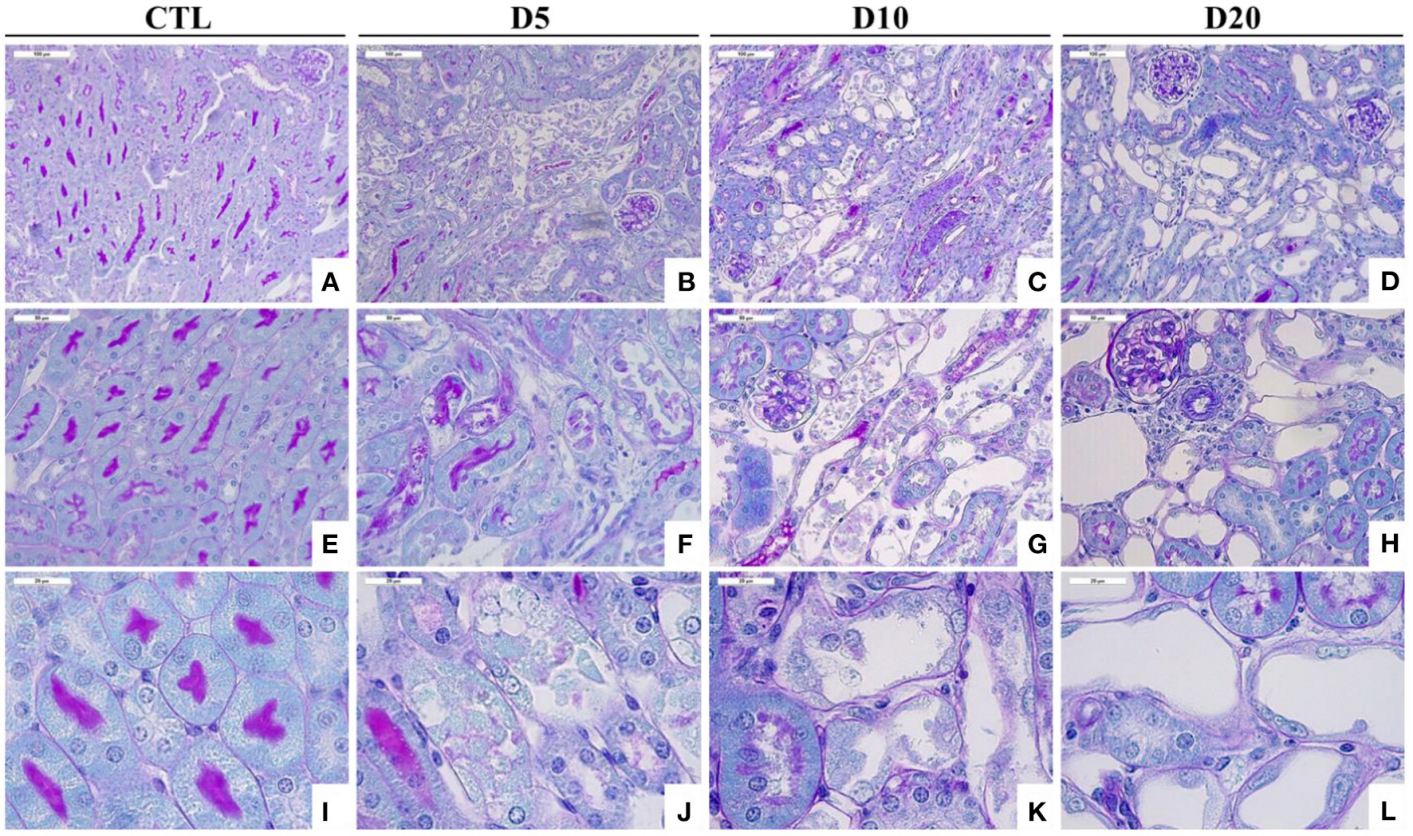
Time course of histological alterations in experimental AAN. Representative photographs of hemalun, Luxol fast blue and Periodic Acid Schiff stained kidney sections [x200 **(A–D)**, x400 **(E–H)**, and x1000 **(I–L)**] from CTL mice and mice intoxicated with AA (aristolochic acid I, Sigma-Aldrich, St. Louis, MO, USA) during 4 consecutive days. Mice were sacrificed 5, 10, and 20 days after first day of AA treatment. Necrotic tubules with cell debris in tubular lumen are visible in mice treated with AA at days 5 and 10 and cystic tubules are visible in mice at days 10 and 20.

The selective toxicity of AA for PTEC has rapidly been highlighted (26). In our Wistar rat model, structural and functional parameters of proximal tubular injury were longitudinally investigated: (1) the acute tubular necrosis histologically delineated to the S3 segment (from day 1 to day 5 after the beginning of AA subcutaneous exposure) was found concomitant to a dramatic urinary increase in low molecular weight proteins, reflecting the loss of reabsorptive capacity by the apical brush border of the tubular epithelium; (2) consecutively to interstitial infiltration by inflammatory cells, areas of non-regenerative tubular necrosis were rapidly replaced by severe tubular atrophy and were surrounded by interstitial fibrosis. In addition, in the rat Wistar model, induction of active caspase-3 in the PTEC demonstrated the contribution of apoptosis in tubular atrophy (27). This biphasic evolution was reproduced in our mouse models (Figure 2). The PTEC targeting by AA suggested the involvement of specific molecular mechanisms that could be responsible for the accumulation of the toxin in these cells. This hypothesis has been confirmed *in vitro* (32, 33) and *in vivo* (28, 34) by several studies reporting a critical role of the organic anion transporter (OAT) family in AA uptake into the PTEC. Specifically, we demonstrated that in AA-treated mice, probenecid treatment reduced tubular necrosis, lymphocytic infiltrate, tubular atrophy as well as fibrosis by blocking AA entry into PTEC as attested by the reduction of DNA-adducts formation (28).

Moreover, it has been pointed out that the formation of AA-DNA adducts is not necessarily associated to AA nephrotoxicity (35, 36). The formation of those adducts is not the only source of cytotoxicity. Indeed, AA-induced cytotoxicity could also be linked to the release of Ca^2+^ from the endoplasmic reticulum (ER) that causes ER and mitochondrial stress resulting in caspases activation and apoptosis (37–39). Furthermore, we and others reported that AA led to an increase in oxidative stress that contributed to DNA damage and cell cycle arrest in experimental studies (27, 40–44). In this regard, Bonventre demonstrated that following AA-induced AKI, PTEC stop their cell cycle in the G2/M phase. This arrest is considered as a protective mechanism as it could help to repair DNA lesions (44). However, this mechanism has also been found to be associated with an excessive production of transforming growth factor β (TGFβ) and connective tissue growth factor (CTGF) thereby promoting fibrosis.

The cell death process (necrosis, apoptosis or autophagy) may also influence the outcome of AKI. Indeed, PTEC necrosis yields pro-inflammatory factors that act as dangers associated molecular patterns (DAMPs). After linking with Toll like receptors (TLR), DAMPs lead to an activation of the innate immune system and a pro-inflammatory environment (45). On the other hand, some authors postulate that apoptosis does not constitute a maladaptive process but is rather a way to eliminate the damaged cells without the release of cellular debris. This hypothesis is supported by the study of Bonventre who demonstrated reduced apoptosis in the AAN model as compared to other AKI models such as IRI. AAN animals also displayed more extensive fibrosis, suggesting that apoptosis constitutes an adaptive mechanism and that cells undergoing cell cycle arrest instead of progressing to apoptosis exert profibrogenic effects (46, 47). However, the role of p53 inhibition on chronic lesions remains controversial: some authors described a worsening of the lesions (48) as others suggested the opposite (49). Recently, the impact of p53 on AAI-induced nephrotoxicity and DNA damage was investigated *in vivo* using *Trp53(*+*/*+*), Trp53(*+*/–), and Trp53(–/–)* mice (50). Interestingly, renal injury was more severe in AAI-treated *Trp53(–/–)* mice relative to *Trp53(*+*/*+*)* and *Trp53(*+*/–)* mice (50).

Finally, we performed an analysis of urine metabolites from rats intoxicated with AA by the use of NMR spectrometry. A significant reduction in Krebs cycle components (α-cetoglutarate, succinate, citrate) was found, suggesting a mitochondrial injury (51).

Finally, PTEC could also contribute to interstitial fibrosis through a complex phenomenon called epithelial-to-mesenchymal transformation (EMT) (see Section The Myofibroblasts) (27, 52).

### The Endothelial Cell Injury

The preservation of endothelium structure and function constitutes a key element of renal function. In this regard, numerous kidney injury models have demonstrated a sustained renal vasoconstriction and a rarefaction of the peritubular capillaries (PC) around injured tubules as prominent features of kidney disease (53) ultimately leading to renal hypoxia thereby promoting fibrosis and progression to CKD (54–57).

Following injury, renal endothelial cells switch from a quiescent to an activated state, leading to an imbalance in the production of vasoactive substances inducing a vasoconstrictive environment and a reduced blood flow. Specifically, a decreased bioavailability of nitric oxide (NO) was described as a characteristic feature of kidney disease (58).

Moreover, a long-term consequence of AKI is a PC rarefaction that is also considered as a hallmark of CKD (31, 59, 60). This PC rarefaction is thought to be due to an anti-angiogenic environment (60) and to the loss of endothelial cells as well as pericytes that transdifferentiate into myofibroblasts (see section Conclusions). It also emphasizes the fact that endothelial cells, unlike tubular cells, fail to regenerate (61, 62).

Only few studies have addressed the involvement of the vascular network in AAN pathophysiology. In this regard, Depierreux et al. were the first to describe a thickening of the walls of interlobular and afferent arterioles due to swelling of endothelial cells in human kidney biopsies from Belgian AAN patients. They proposed that primary lesions could occur in the vessel walls, then inducing tubular destruction (12). Since then, other investigators demonstrated a dramatic decrease in PC, in particular in the fibrotic areas in human renal tissue samples as well as in experimental models (63, 64). In a rat model of AAN, the reduction of PC network was associated to a decreased expression of vascular endothelial growth factor (VEGF) and to an increased expression of hypoxia inducible factor 1α (HIF-1α), thereby suggesting that ischemia and hypoxia are critical processes contributing to AAN progression (63). Similar data were also reported by Wen et al. in a rat model of AAN. Interestingly, they also described an imbalance between the vasoactive factors with a reduced NO production occurring along with an increase in mRNA and protein expression of endothelin (ET) (65). Such imbalance between vasoactive substances was also investigated in our lab in a mouse model of AAN. In our hands, renal NO bioavailability was reduced following AA intoxication not only during the acute phase (66) but also during the progression to the chronic stage (29). We also highlighted that oral treatment with L-Arginine (L-Arg) led to the maintenance of renal NO bioavailability along with a reduction of AA toxicity (29, 66). Several studies have reported the impact of AA on endothelial cells in the literature. Shi and Feng showed that AA decreased cell viability in a dose- and time-dependent manner. They also demonstrated that AA could induce endothelial cell apoptosis and increase activation of caspase-3 (67). Guan et al. highlighted that HUVEC treated with AA display impaired angiogenesis capacities (68). More recently, our team showed a cytotoxic effect of AA on EAhy926 endothelial cells. In addition, exposure of aortic rings to AA impaired vascular relaxation to acetylcholine (69).

### The Role of the Immune System

The histological examination of renal tissue samples from the first human cases had suggested that AAN was characterized by a diffuse and pauci-cellular interstitial fibrosis along with a corticomedullary gradient and marked tubular atrophy (12). The involvement of the immune system in AAN pathophysiology was rapidly evoked thanks to the results of a pilot clinical study in which steroids treatment resulted in slowing down the progression of the renal failure (70, 71). In addition, histopathological analyses from AAN patients highlighted the presence of an inflammatory infiltrate of mixed origin (monocytes/macrophages, B-lymphocytes and CD8 cytotoxic T-cell (72) or mastocytes (73), leading to a paradigm shift regarding the role of interstitial inflammation in AAN. Indeed, the role of the immune system has been largely demonstrated in both ischemic and other toxic AKI models (74).

In our AAN Wistar model, a significant inflammatory infiltrate following acute tubular necrosis has been described (27, 30). During the acute phase, injured PTEC secrete inflammatory cytokines contributing to the accumulation of inflammatory cells into the renal interstitium (increased urinary levels of MCP-1 on day 7 and of IL-1α on day 10). During the chronic phase (day 35), a significant urinary release of IL-1α, TNF-α, IFN-γ, MCP-1, IL-4, and TGFβ was observed (27, 30). Moreover, an increased cortical expression of hyaluronan (HA) following tubular necrosis has been demonstrated in our AA-intoxicated mice as in other AKI models such as the IRI model (75–77). This ubiquitous glycosaminoglycan is normally found in the extracellular matrix of the renal medulla, but not the cortex, except during inflammation and ischemic injuries (76, 78). In inflammatory conditions, HA can be cleaved by hyaluronidases, generating low molecular weight “fragments” that interact with TLR2 and 4 (77, 79) thereby contributing to an inflammatory environment (76, 77, 79).

Following tubular necrosis and cytokines secretion, an inflammatory infiltrate including various immune cells such as dendritic cells (DC), neutrophils, macrophages, natural killer T-cells (NKT) and T and B lymphocytes has been described in AKI (74). The respective functions of these cells remain controversial, depending on the AKI model and the time course of the disease. Regarding experimental AAN, the role of the different immune cell types was investigated only recently. Macrophages surrounding injured PTEC were observed in our AA-intoxicated rats and mice models (27, 29, 30). Phagocytosis of cell debris may lead to macrophage maturation (MHC class II expression) and stimulation of naive lymphocytes. The macrophage infiltrate was rapidly followed by a lymphocytic infiltrate composed of CD8^+^ and CD4^+^ T-cells (80). Using several depletion protocols (anti-CD4^+^, anti-CD8^+^ or anti-CD25^+^) in our AAN mouse model, we investigated the respective roles of these specific cells. We observed an aggravation of AA-induced AKI in mice depleted with anti-CD4^+^ or anti-CD8^+^ T-cells along with an increased *TNF-*α and *MCP-1* mRNA renal expression. However, regulatory T-cells depletion did not modify the severity of AKI, suggesting an independent mechanism (80). An increased proportion of myeloid CD11b^high^F4/80^mid^ and a decreased proportion of their counterpart CD11b^low^F4/80^high^ population was also observed after AA intoxication. After CD4^+^ T-cell depletion, the increase in the CD11b^high^F4/80^mid^ population was even higher whereas the decrease in the CD11b^low^F4/80^high^ population was more marked after CD8^+^ T-cell depletion. These results suggest that CD4^+^ and CD8^+^ T-cells are able to limit the severity of AA-induced AKI. The protective effect of CD4^+^ and CD8^+^ T-cells has been associated with an imbalance of the CD11b^high^F4/80^mid^ and CD11b^low^F4/80^high^ populations (80).

### The Myofibroblasts

Tubulointerstitial fibrosis constitutes the final common pathway of CKD. In this regard, myofibroblasts are commonly pointed as the predominant effector cells of this process. They represent a unique population of cells that appears *de novo* after kidney injury due to the secretion of factors by injured tubules and inflammatory cells (31, 81). They exert their pro-fibrotic function by producing the extracellular matrix, the crosslinking enzymes and the inhibitors of matrix degrading metalloproteinases (52). Expression of αSMA, a stress fiber protein that facilitates increased contractility, along with a typical fibroblastic morphology is their defining feature (52).

Given their pivotal importance, the cellular origin of those cells constitutes a critical question as well as an ongoing debate. The following cellular origins are proposed:

- Interstitial fibroblasts are commonly regarded as the most abundant progenitor to myofibroblasts (31).
- Proximal epithelial tubular cells (PTEC) were historically identified as a potential source of myofibroblasts. A process called epithelial-to-mesenchymal transition (EMT) was identified as an important mechanism responsible for the accumulation of interstitial myofibroblasts and collagen production during kidney fibrosis. Many *in vitro* studies have described the expression of mesenchymal markers when epithelial cells are injured. However, studies supporting the existence of EMT *in vivo* in the kidney have been much more limited and this hypothesis is nowadays considered as unlikely to occur *in vivo* (82). It is now proposed that expression of mesenchymal markers by epithelial cells *in vivo* should rather be interpreted as a sign of dedifferentiation (83).
- Endothelial cells (EC) have also been proposed as progenitors for myofibroblasts. A process called endothelial-to-mesenchymal transition (endo-MT) was described as a trans-differentiation of EC to a more mesenchymal phenotype. The magnitude of endo-MT contribution to the myofibroblast pool is variable depending on the experimental model used (52).
- Pericytes are specialized cells found in close vicinity to EC. Their role consists in maintaining vascular stability via cell-cell communication and by the release of factors such as TGFβ or VEGF (83). It is now proposed that renal pericytes could migrate from perivascular location to the interstitium and then acquire the phenotype of the myofibroblast. Nowadays, pericytes receive much interest since targeting these cells could lead to preservation of vascular network along with inhibition of fibrosis (83).
- Fibrocytes are circulating cells of myeloid lineage that are proposed to be the precursors of fibroblasts (83). These cells, deriving from the bone marrow, do not appear as major contributors to the pool of myofibroblasts in the kidney. It is rather proposed that they may act by paracrine signaling on other kidney cells to support and promote renal scarring (52, 83).

**Figure 3:**
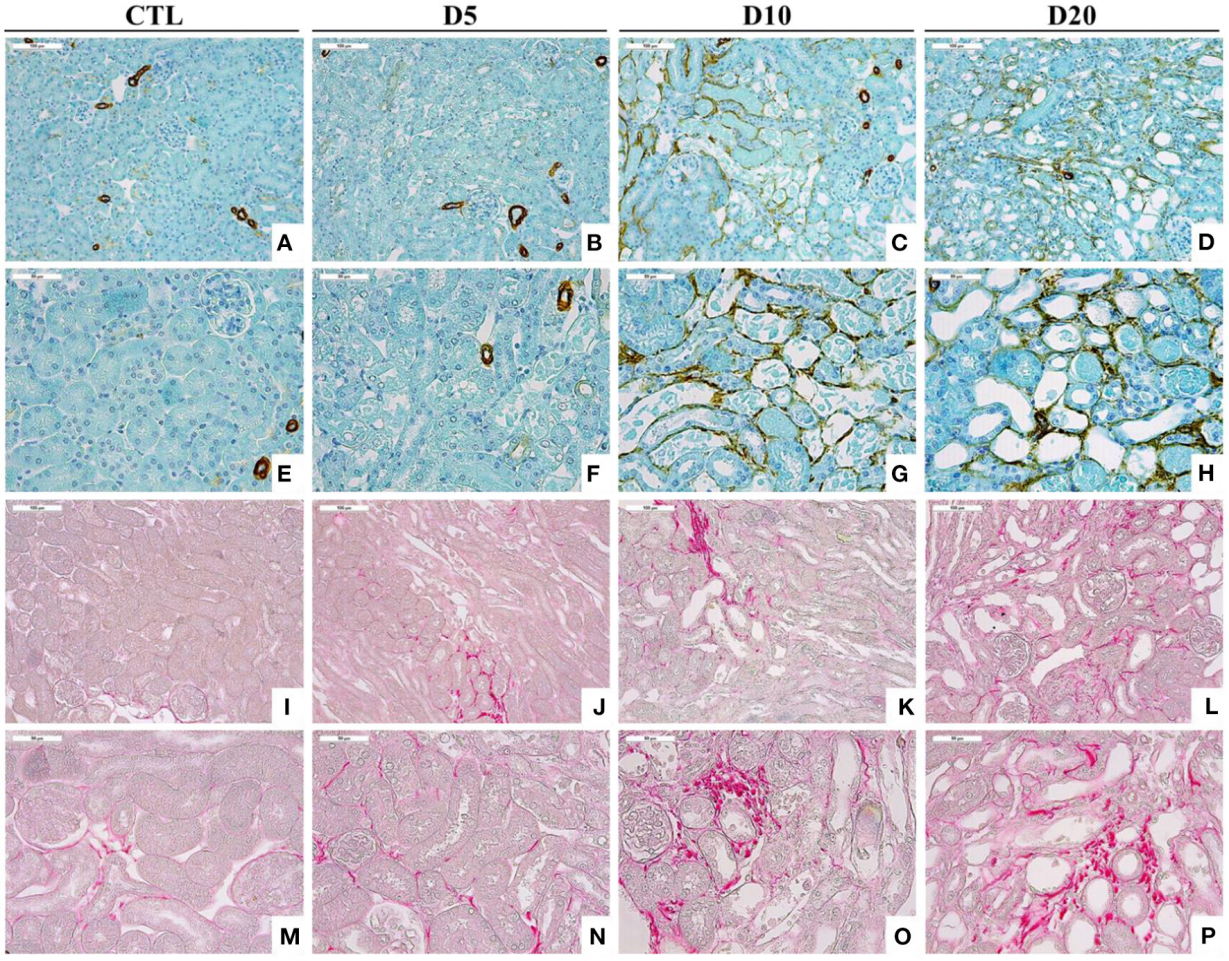
Accumulation of αSMA-positive cells **(A–H)** and collagen I and III, highlighted by Sirius Red staining **(I–P)**, within the interstitium of the kidney of CTL mice and mice intoxicated with AA (aristolochic acid I, Sigma-Aldrich, St. Louis, MO, USA) during 4 consecutive days. Mice were sacrificed 5, 10, and 20 days after first day of AA treatment [Magnification x100 for **(A–D)** and **(I–L)** and x200 for **(E–H)** and **(M–P)**].

In experimental AAN, we have described that myofibroblasts as well as collagen accumulate in the interstitium following AA intoxication (Figure 3) (27, 29). In our Wistar rat model, we have highlighted the loss of the epithelial phenotype (N-cadherin and E-cadherin) along with the acquisition of mesenchymal cell markers (vimentin and αSMA) in PTEC during the acute phase of AAN (27). However, transmembrane migration was not observed leading to the conclusion that EMT was rather unlikely to occur. More recently, we showed that the early inhibition of the p-Smad2/3 signaling pathway by neutralizing anti-TGFβ antibody (1D11) improved renal function impairment and partially prevented epithelial-endothelial axis activation by reducing *platelet derived growth factor receptor* β (*PDGFR*β)^+^ pericytes (84).

## Discussion

Considering the data collected from our initial Wistar rat model and our consecutive mouse models, we were able to relate simultaneous or successive events in relation with the four pivotal cellular responses proposed by Eddy (31) in order to link AKI to CKD mechanisms. The first insult resulting from AA intoxication is the onset of non-regenerative proximal tubular necrosis and early peritubular endothelial insult, resulting in a prominent interstitial influx by immunocompetent cells and hypoxia, respectively. Whatever the duration of AA intoxication (sustained to 35 days in our rat Wistar model or discontinued after 5 or 20 days in our mouse models), the inflammatory response is likely to be considered as the physiopathological link in the progression from the acute phase to the chronic phase, namely AKI-to-CKD transition. The promising attempts in blocking AA entry into PTEC or in modulating targeted subpopulations of immunocompetent cells are clearly attractive strategies to be more deeply investigated in the future in order to enhance recovery from AKI. Furthermore, the sustained impairment of the vascular network and the onset of myofibroblasts, a key cellular population in kidney disease progression, were also reported in our Wistar rat and mice AAN models.

Considering these “adaptative” AAN models as well as the four pivotal cellular responses to a toxic insult, interventional strategies could be interestingly explored, mimicking clinical situations. Indeed, there is no consensus available today regarding the definition of recovery from AKI. Several definitions exist such as those of Acute Dialysis Quality Initiative (ADQI), Kidney Disease: Improving Global Outcomes (KDIGO) and Acute Renal Failure Trial Network (ATN) and they are not unequivocal (85). There is increasing evidence suggesting that AKI and CKD should be considered as an interconnected syndrome (9). Clinical studies and experimental research strongly suggest that even after mild AKI and “apparent” recovery of renal function, clinical subjects or experimental animals are at increased risk of developing CKD (86). In our opinion and according to those from other authors, this strongly suggests that there is no recovery *ad integrum* after AKI and that subclinical lesions (i.e., with normal plasma creatinine measurement) favor the onset of secondary CKD. It is likely that compensatory mechanisms exist such as “compensatory hypertrophy” of the remaining contralateral kidney after nephrectomy (in case of living kidney donor for instance). However, this mechanism cannot be considered as a recovery *ad integrum* even if the kidney function is seemingly normal. Moreover, it is now obvious that repeated “minor” episodes of AKI constitute a springboard to CKD. From that perspective, our AAN models differ from other classical AKI models such as ischemic reperfusion injury (IRI), unilateral ureteral obstruction (UUO), 5/6e nephrectomy model and nephrotoxic AKI models such as ciclosporin, cisplatin, and adriamycin. Indeed, the toxic insult due to multiple AA injections, even if mild, is sustained in time. Therefore, tubular regeneration is dampened, repair is maladaptive especially in the context of DNA damage and severe reduction of peritubular capillaries leading ultimately to tubular atrophy, interstitial chronic inflammation, vascular rarefaction and prominent fibrosis. Each model suffers from technical difficulties and limitations regarding extrapolation to clinical situations. However, we actually would like to underline some advantages of our AAN models: they do not require any particular skills by opposition to surgical models, the toxicity is rapidly induced and it generates homogenous alterations in both kidneys; moreover, contrasting with UUO or unilateral IRI, there is no renal compensation from the other kidney; finally, there is prominent fibrosis in the chronic phases of the model. Despite numerous advantages of the AAN models, it should be mentioned that the genotoxic properties of AA could represent a limitation of the AAN models—but only in chronic exposure (at least more than 3 months in our hands). Indeed, AA have been recognized for many years as a human carcinogen causing upper urinary tract carcinoma but also bladder cancer and renal cell carcinoma (18). Exposure of rodents to AA could interfere with cell cycle of the urothelium or the digestive tract and induce activation of protooncogens such as H-ras. In rodents, H-ras has been found to play the same role as TP53 (14).

## Conclusions

The incidence and prevalence of CKD are still rising worldwide (87, 88). It has become increasingly clear that AKI constitutes a significant risk factor for the development of CKD (9). In this regard, animal models play a crucial role in unraveling the pathological mechanisms of the so-called AKI-to-CKD transition (10). On the basis of our findings as well as data from the literature, we were able to observe a biphasic evolution of the structure and function parameters following AA intoxication with tubular injury which constitutes the first pivotal cellular event. Following PTEC injury, inflammation was described as persisting all along the experimental protocols. We propose that the inflammatory response constitutes the link in the progression from the acute phase to the chronic phase. The prolonged inflammatory response hampered tubular regeneration and ultimately led to tubular atrophy, interstitial chronic inflammation, peritubular capillaries rarefaction and prominent fibrosis. Finally, impairment of the vascular network and myofibroblasts, a key cellular population in kidney disease progression, were also described in our AAN rodent models. In conclusion, these models recapitulate the four pivotal cellular responses that are crucial in the AKI-to-CKD transition.

## Author Contributions

BT and JI wrote together the manuscript and prepared the figures. DA-E, AM-H, BO, DE, PA, and HC reviewed the draft and contributed to its improvement. CJ-M, CN, and JN supervised each step of the process, including the selection of the bibliographical references. All authors contributed to the article and approved the submitted version.

## Conflict of Interest

The authors declare that the research was conducted in the absence of any commercial or financial relationships that could be construed as a potential conflict of interest.

## Publisher's Note

All claims expressed in this article are solely those of the authors and do not necessarily represent those of their affiliated organizations, or those of the publisher, the editors and the reviewers. Any product that may be evaluated in this article, or claim that may be made by its manufacturer, is not guaranteed or endorsed by the publisher.
